# Children's Enrollment in Children's Health Insurance Program (CHIP) Coverage During the Medicaid Unwinding

**DOI:** 10.1111/1475-6773.70078

**Published:** 2025-12-19

**Authors:** Erica L. Eliason, Daniel B. Nelson, Aditi Vasan

**Affiliations:** ^1^ Center for State Health Policy, Rutgers University New Brunswick New Jersey USA; ^2^ Department of Urban‐Global Public Health Rutgers School of Public Health Newark New Jersey USA; ^3^ Division of General Internal Medicine Oregon Health & Science University Portland Oregon USA; ^4^ PolicyLab, Children's Hospital of Philadelphia Philadelphia Pennsylvania USA; ^5^ Department of Pediatrics University of Pennsylvania Perelman School of Medicine Philadelphia Pennsylvania USA

**Keywords:** child health services, children's health, children's health insurance program, Medicaid, state health policy

## Abstract

**Objective:**

To examine changes in children's Medicaid and CHIP enrollment during the Families First Coronavirus Response Act unwinding and assess whether CHIP enrollment offsets Medicaid declines.

**Study Setting and Design:**

We used linear probability models with monthly indicators to estimate changes in enrollment from April 2023 to September 2024 overall and by CHIP structure type.

**Data Sources and Analytic Sample:**

We used monthly children's enrollment data from the U.S. Centers for Medicare & Medicaid Services for 32 states and the District of Columbia.

**Principal Findings:**

During the unwinding, Medicaid enrollment declined from 48.1% to 41.2% of children, while CHIP enrollment remained stable (8.7% to 8.6%). We found average declines of 62,032 (95% confidence interval [CI]: −108,018 to −16,045) Medicaid‐enrolled children per state (6.5 percentage points [pp], 95% CI: −8.1 to −5.0). Medicaid declines were larger in states with combination CHIP (−8.7 pp, 95% CI: −10.3 to −7.2) than Medicaid expansion CHIP (−4.5 pp, 95% CI: −6.0 to −3.1). We found no evidence of significant changes in CHIP enrollment overall or by CHIP structure.

**Conclusions:**

Children's Medicaid enrollment fell sharply without offsetting CHIP gains during the unwinding, underscoring the need for policies that prevent administrative disenrollment and ensure seamless coverage transitions.

## Callout Box

- What is known on this topic
  ○ The Families First Coronavirus Response Act (FFCRA) continuous coverage provision prevented Medicaid disenrollment during the COVID−19 public health emergency, improving coverage stability for children.
  ○ Projections estimated that millions of children could shift from Medicaid to CHIP during the rollback of the FFCRA (the “unwinding”), despite overall projected declines in Medicaid and CHIP enrollment.
  ○ Prior research found overall declines in children's public coverage during the unwinding, with larger reductions in states with separate CHIP components, but did not disaggregate Medicaid and CHIP enrollment.
- What this study found
  ○ During the first 17 months of the FFCRA unwinding, children's Medicaid enrollment declined substantially (−6.5 percentage points [pp] on average) with no significant changes in CHIP enrollment.
  ○ Declines in children's Medicaid were larger in combination CHIP states (−8.7 pp) than in Medicaid expansion CHIP states (−4.5 pp), yet CHIP enrollment remained stable in both sets of states.
  ○ Findings suggest that millions of children lost Medicaid without transitioning to CHIP, highlighting the need for policies to facilitate seamless coverage transitions.

## 1 Introduction

The Children's Health Insurance Program (CHIP) was designed to create a coverage option for children in low‐income families whose incomes were too high to qualify for Medicaid but who could not afford private coverage [1]. CHIP has been found to be an important mechanism for covering children, providing insurance to over 7 million enrollees as of 2024, and improving access to care and children's health outcomes [2, 3]. States structure their CHIP coverage in one of three ways: (1) expansions of their Medicaid programs (Medicaid expansion CHIP, or M‐CHIP), (2) CHIP coverage that is entirely separate from Medicaid (separate CHIP), or (3) a combination of both approaches (combination CHIP) [4]. States with M‐CHIP provide Medicaid to CHIP enrollees and follow federal Medicaid rules, while states have greater flexibility over separate CHIP. States with combination CHIP provide M‐CHIP for some groups (e.g., by age or income) and separate CHIP for others.

To support health care access during the COVID‐19 public health emergency, the March 2020 Families First Coronavirus Response Act (FFCRA) included the continuous coverage provision, which offered enhanced federal matching funds to states for the continuous enrollment of Medicaid beneficiaries [5]. As a result, Medicaid enrollees retained continuous coverage for the duration of this FFCRA provision. This provision also applied to M‐CHIP, but did not automatically apply to separate CHIP, including separate CHIP coverage in combination CHIP states [6]. However, some states opted to provide continuous coverage in separate CHIP using state‐only funds. All states had the option to submit a COVID‐19 Section 1115 waiver to CMS that would allow them to cover CHIP enrollees [6].

As of April 2023, states were allowed to begin the FFCRA “unwinding” process, which involved resuming Medicaid eligibility redeterminations and disenrollments and phasing out the FFCRA continuous coverage protections [5]. During the unwinding, in states that had separate CHIP components, children who were no longer eligible for Medicaid but remained eligible for CHIP could have transitioned to separate CHIP coverage, given its higher income eligibility thresholds [7]. Projections estimated that millions of children could shift from Medicaid to separate CHIP during the unwinding, despite overall projected declines in Medicaid and CHIP enrollment [8].

Research found that the unwinding led to declines in children's total Medicaid and CHIP enrollment, with larger reductions in states that included separate CHIP components compared to M‐CHIP states [9]. However, this previous study did not distinguish between Medicaid and CHIP enrollment, which may have missed CHIP's potential to cover some children transitioning from Medicaid during the unwinding [9]. Thus, the objective of this study was to examine children's coverage during the FFCRA unwinding, focusing on the extent to which changes in CHIP enrollment offset declines in Medicaid enrollment.

## 2 Methods

### 2.1 Data and Study Population

This study used state reports of monthly U.S. Centers for Medicare & Medicaid Services (CMS) enrollment data in Medicaid and CHIP [10, 11]. Separate CHIP enrollment data was originally collected starting on April 1, 2023 as states were required to submit separate CHIP enrollment to CMS to fulfill reporting requirements under section 1902(tt) [1] of the Social Security Act [10] (described in Appendix A1). In states that offer perinatal CHIP using the same CHIP type as children, we were unable to distinguish perinatal enrollees from child enrollees in the data. We therefore excluded 16 states where child and perinatal CHIP enrollment could not be separated. We additionally excluded Arizona, which did not report children's enrollment, and Maryland, which changed their CHIP policies during the study [10]. After these exclusions, the study sample included a total of 32 states and the District of Columbia (DC), which were generally representative of children's national eligibility limits (Appendix A2). Our study states captured the majority of M‐CHIP states and half of combination CHIP states, but did not include the 2 states with only separate CHIP (Appendix A2). This study using publicly available data was not considered to be human subjects research by the Rutgers University institutional review board.

### 2.2 Measures

Our outcomes of interest were children's enrollment in Medicaid and CHIP. We measured children's enrollment as both the number of enrolled children and as a proportion of a state's child population, using population data from the U.S. Census Bureau.

We examined state CHIP and Medicaid characteristics and unwinding policies, including CHIP structure type for children's coverage, an indicator for which month the state began the unwinding, the unwinding strategy for prioritizing renewals (time‐based, state‐determined approach, or hybrid approach), estimated time to complete renewals (less than 9 months, 9 to less than 12 months, 12–14 months), policies for flagging potentially ineligible enrollees, policies for automated transfer to separate CHIP if Medicaid ex parte review confirmed CHIP eligibility, and the number of section 1902(e)(14) waivers to address challenges related to Medicaid unwinding [12, 13, 14].

### 2.3 Study Design and Statistical Analysis

We estimated linear probability models to examine changes in children's enrollment during the unwinding. The models included fixed effects for monthly indicators (0–17) to capture enrollment changes. We focused on differences in September 2024 (month 17) relative to April 2023 (month 0), the start of the unwinding period, to estimate changes in enrollment since the unwinding began. We estimated changes in children's enrollment overall and separately by CHIP structure type for children's coverage. To account for differences across states and policy factors, we adjusted for state fixed effects, state CHIP and Medicaid characteristics, and unwinding policies. Standard errors were clustered at the state level to account for within‐state correlation and the non‐independence of repeated observations over time. As a sensitivity analysis, we estimated models weighted by states' child population size using data from the U.S. Census Bureau.

## 3 Results

Among the 32 study states and DC, most study states had M‐CHIP structures (19%, or 57.6%), began the unwinding in May 2023 (13%, or 39.4%), used state‐determined approaches for prioritizing renewals (13, or 39.4%), and had an estimated time to complete renewals of 12–14 months (29%, or 87.9%) (Table 1). Approximately half had policies for flagging potentially ineligible enrollees (16%, or 48.5%), and 10 states (30.3%) had automated transfers to separate CHIP from Medicaid ex parte reviews. On average, states had 8 section 1902(e)(14) waivers.

**TABLE 1:** State unwinding policies and children's health insurance program (CHIP) and Medicaid characteristics, 2023.

Characteristics	Overall
CHIP structure type, *N* (%)	
Medicaid expansion CHIP	19 (57.6)
Combination CHIP	14 (42.4)
First month of Medicaid unwinding, *N* (%)	
April 2023	4 (12.1)
May 2023	13 (39.4)
June 2023	10 (30.3)
July 2023	4 (12.1)
August 2023	2 (6.1)
Unwinding strategy for prioritizing renewals, *N* (%)	
Time‐based approach	7 (21.2)
Population‐based approach	1 (3.0)
State‐determined approach	13 (39.4)
Hybrid approach	11 (33.3)
Not recorded	1 (3.0)
Estimated time to complete renewals, *N* (%)	
9 to less than 12 months	3 (9.1)
12 to 14 months	29 (87.9)
Not recorded	1 (3.0)
Flagging potentially ineligible enrollees, *N* (%)	
Yes	16 (48.5)
No	16 (48.5)
Not recorded	1 (3.0)
Automated transfer to separate CHIP from Medicaid ex parte review, *N* (%)	
Yes	10 (30.3)
N/A	19 (57.6)
Not recorded	4 (12.1)
Average Number of Section 1902(e)(14) Waivers	8.0

**FIGURE 1:**
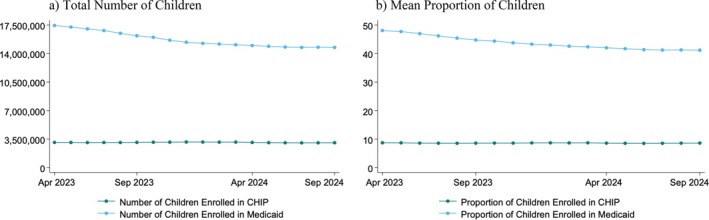
Trends in children's Medicaid and CHIP enrollment during the FFCRA Medicaid unwinding, 4/2023–9/2024. (a) total number of children (b) mean proportion of children. *Note:* Authors' analysis of U.S. Centers for Medicare & Medicaid Services enrollment data in Medicaid and CHIP, April 2023 to September 2024. CHIP is the Children's Health Insurance Program. FFCRA is the Families First Coronavirus Response Act.

During the unwinding, the total number of children enrolled in Medicaid in the study states declined from 17,440,324 enrollees in April 2023 to 14,748,095 Medicaid‐enrolled children in September 2024 (Figure 1a). Over the same period, CHIP enrollment remained relatively stable, with 3,090,573 children enrolled in April 2023 and 3,056,265 in September 2024. As a share of the population, Medicaid enrollment declined from 48.1% of children in April 2023 to 41.2% in September 2024, while CHIP enrollment was relatively stable, at 8.7% in April 2023 and 8.6% in September 2024 (Figure 1b).

In adjusted models, states had on average 62,032 fewer Medicaid‐enrolled children (95% CI: −108,018 to −16,045) in September 2024 than in April 2023, with no statistically significant change in CHIP enrollment (740 fewer children on average; 95% CI: −10,815 to 9336) (Table 2; full regression results for main overall adjusted models listed in Appendix A3). The average proportion of Medicaid‐enrolled children declined by 6.5 percentage points (95% CI: −8.1 to −5.0), while changes in CHIP enrollment proportions were not statistically significant (−0.5 percentage points; 95% CI: −1.4 to 0.5).

**TABLE 2:** Changes in children's Medicaid and CHIP enrollment during the FFCRA Medicaid unwinding overall and by CHIP structure type, 4/2023–9/2024.

Outcome	Start of FFCRA medicaid unwinding, April 2023	17 months into medicaid unwinding, September 2024	Unadjusted change (95% CI)	*p*	Adjusted change (95% CI)	*p*
Overall						
Average number of children enrolled in CHIP	93,654	92,614	−1040 (−10,394 to 8315)	0.822	−740 (−10,815 to 9336)	0.882
Average number of children enrolled in Medicaid	528,495	446,912	−81,583 (−130,422 to −32,743)	0.002	−62,032 (−108,018 to −16,045)	0.01
Average proportion of children enrolled in CHIP	8.7	8.6	−0.1 (−0.8 to 0.6)	0.765	−0.5 (−1.4 to 0.5)	0.327
Average proportion of children enrolled in Medicaid	48.1	41.2	−6.9 (−8.2 to −5.5)	< 0.001	−6.5 (−8.1 to −5.0)	< 0.001
States with M‐CHIP structures						
Average number of children enrolled in CHIP	85,269	81,131	−4139 (−14,792 to 6515)	0.425	−5810 (−16,064 to 4444)	0.249
Average number of children enrolled in Medicaid	423,977	379,132	−44,844 (−73,345 to −16,344)	0.004	−30,542 (−52,652 to −8433)	0.009
Average proportion of children enrolled in CHIP	8.8	8.5	−0.3 (−1.3 to 0.7)	0.583	−0.7 (−1.8 to 0.4)	0.198
Average proportion of children enrolled in Medicaid	48.4	43.3	−5.1 (−6.4 to −3.7)	< 0.001	−4.5 (−6.0 to −3.1)	< 0.001
States with Combination CHIP Structures						
Average number of children enrolled in CHIP	105,033	108,199	3166 (−15,633 to 21,965)	0.722	3226 (−16,057 to 22,509)	0.724
Average number of children enrolled in Medicaid	670,340	538,898	−131,442 (−245,077 to −17,806)	0.03	−102,518 (−200,830 to −4206)	0.04
Average proportion of children enrolled in CHIP	8.6	8.7	0.1 (−1.1 to 1.3)	0.849	−0.4 (−2.4 to 1.6)	0.671
Average proportion of children enrolled in Medicaid	47.6	38.3	−9.3 (−11.5 to −7.2)	< 0.001	−8.7 (−10.3 to −7.2)	< 0.001

*Note:* Authors' analysis of U.S. Centers for Medicare & Medicaid Services enrollment data in Medicaid and CHIP, April 2023 to September 2024. CHIP is the Children's Health Insurance Program. FFCRA is the Families First Coronavirus Response Act.

Among combination CHIP states, declines in both the number (102,518 fewer children; 95% CI: −200,830 to −4206) and proportion (−8.7 percentage points; 95% CI: −10.3 to −7.2) of Medicaid‐enrolled children were larger than in M‐CHIP states (30,542 fewer children; 95% CI: −52,652 to −8433; −4.5 percentage points; 95% CI: −6.0 to −3.1). There were no significant changes in the number or proportion of children enrolled in CHIP during the unwinding in states with combination CHIP or M‐CHIP. In sensitivity analyses weighted by population, effect sizes for the overall declines in children's Medicaid enrollment were larger, but all estimates had confidence intervals that overlapped with those in the main models (Appendix A4).

## 4 Discussion

Using CMS enrollment data on children's Medicaid and CHIP from the first 17 months of the FFCRA unwinding, we found significant declines in children's Medicaid enrollment, with no corresponding changes in CHIP. Consistent with prior research showing larger reductions in children's public coverage in states with combination or separate CHIP structures than in M‐CHIP states, we observed smaller declines in children's Medicaid t in M‐CHIP states [9]. Across all states and CHIP structures, we found no evidence of significant changes in children's CHIP enrollment during the unwinding. In sensitivity analyses weighted by population, the larger point estimates for the decline in the number of Medicaid‐enrolled children during the unwinding suggest that states with larger child populations experienced greater reductions in enrollment. However, the estimates for the change in the proportion of Medicaid‐enrolled children were similar to the main models, suggesting that proportionate effects were consistent across states regardless of population size.

Federal analyses predicted that approximately three‐quarters of the children who lost Medicaid during the unwinding would lose coverage due to administrative reasons, such as paperwork or procedural barriers, rather than actual changes in their eligibility, leading to unnecessary coverage loss [15]. These projections hypothesized that children in states with separate CHIP components would face greater administrative barriers as M‐CHIP is integrated with Medicaid while separate CHIP is a distinct program, and states therefore need policies in place to automatically transfer enrollees to separate CHIP when they lose Medicaid [15]. Historically, children in states with separate CHIP components have had higher rates of churn between Medicaid and CHIP [16]. In contrast, children in M‐CHIP states were expected to transition more easily from Medicaid to CHIP [15]. In anticipation of the unwinding, some states even converted to M‐CHIP to facilitate coverage transitions [5]. However, our results show no evidence of significant CHIP transitions in either set of states.

Despite projections that millions of children would shift from Medicaid to CHIP as states resumed disenrollment, our findings suggest that millions of children lost Medicaid without accompanying CHIP enrollment gains [8]. Other estimates found that only 10.3% of children's Medicaid enrollment declines were offset by CHIP gains, and that marketplace enrollment gains for children replaced only about 14% of the combined Medicaid/CHIP declines [17]. Recent research found that during the unwinding, Medicaid coverage for children's emergency department visits declined, with a 12.5% increase in the share of visits by children with commercial coverage and a 45.2% increase in the share of visits by uninsured children [18]. These patterns suggest a shift away from Medicaid coverage for children's care towards commercial insurance and, more concerningly, towards higher rates of uninsurance [18]. While our data cannot examine whether children who lost Medicaid transitioned to private coverage or became uninsured, these recent findings suggest that many children may have become uninsured as a result of the unwinding [18], consistent with national reports showing increases in childhood uninsurance and decreases in public coverage in 2023 [19]. These coverage losses could have broad implications nationally as Medicaid and CHIP together cover 37 million children in the U.S., or just under half of all children [20]. States increased outreach and communication efforts during the unwinding, which will be important to maintain to re‐enroll eligible children or connect them to other coverage [21].

Consistent health insurance is essential for children's access to and receipt of high‐quality health care. Children with coverage gaps are significantly more likely to lack a usual source of care, have no well‐child visits, and experience unmet medical or prescription drug needs [22]. Continuous coverage during the FFCRA reduced these coverage gaps, decreased unmet health care needs, and lessened administrative burdens for Medicaid‐enrolled children [23]. These gains are at risk of reversal with Medicaid loss during the unwinding, potentially worsening children's access to care. The unwinding also likely exacerbated inequities in children's coverage stability. Medicaid‐enrolled children are disproportionately children of color [24], and FFCRA‐related improvements in coverage and care were most substantial for Hispanic children [25]. Consequently, the unwinding may have put these groups at the highest risk of coverage loss, further widening coverage disparities [26].

This study covered the period from April 2023 to September 2024. As the poverty rate remained largely stable between 2023 and 2024, it is unlikely that there were substantial changes in children's Medicaid or CHIP income‐based eligibility during this period [27]. During this time, the unwinding was completed in nearly all states, with only Alaska (2025), the District of Columbia (2025), and North Carolina (November 2024) projecting completion dates later than September 2024 [12]. As a result, our estimates likely reflect a nearly complete picture of changes in children's Medicaid and CHIP enrollment among study states during the unwinding. In addition, this period includes 9 months of a new January 2024 federal policy that offers 12 months of continuous eligibility in Medicaid and CHIP for children under the Consolidated Appropriations Act (CAA) of 2023 [28]. This policy, implemented during the unwinding, likely helped reduce some Medicaid loss for children [29]. States could offer 12‐month continuous eligibility for children since 1997 [30], which reduces coverage gaps and improves children's health outcomes [31]. Prior to the CAA, 26 states had adopted this policy in both Medicaid and CHIP, and 7 states had adopted it only in CHIP [5]. Under the CAA, an additional 24 states will newly implement continuous eligibility in Medicaid and 17 in CHIP, potentially improving coverage stability for millions of children [32].

States also had the option to pursue multi‐year continuous eligibility, allowing children to retain Medicaid and CHIP for periods beyond 12 months. As of 2025, nine states were approved for multi‐year continuous eligibility, in some cases providing uninterrupted coverage from birth to age six [33]. However, CMS recently announced that it does not anticipate approving new multi‐year waivers or renewing existing ones, effectively phasing out this approach to keeping children covered for longer periods and limiting states' ability to guarantee continuous Medicaid and CHIP coverage in early childhood [33]. This change could increase the risk of coverage instability for young children, particularly as states continue to grapple with enrollment disruptions following the unwinding.

## 5 Limitations

This study had several limitations. First, our analysis only includes data from April 2023 to September 2024. We did not examine changes relative to the pre‐ or during‐FFCRA periods as data necessary for this analysis were only available starting during the unwinding. While two states and DC were still completing the unwinding during this timeframe, our estimates likely reflect a nearly complete picture of changes among study states, though results could shift slightly as these jurisdictions finish their renewals. Secondly, data were reported at the state‐month level and do not follow individual children, limiting our ability to examine children's coverage trajectories or specific subgroups, such as by race and ethnicity. As a result, the stable CHIP enrollment rates may have masked transitions from Medicaid to CHIP if similar numbers concurrently transitioned from CHIP to other coverage. Finally, our data cannot determine whether children losing Medicaid transitioned to private coverage or became uninsured.

## 6 Conclusion

We found that during the FFCRA unwinding, children's Medicaid enrollment declined substantially without corresponding increases in CHIP, regardless of state CHIP structure. Given Medicaid and CHIP's central role in covering nearly half of U.S. children, these losses may have far‐reaching implications for children's access to care and health outcomes. Policymakers should consider strategies to prevent administrative disenrollment, facilitate seamless coverage transitions, and protect policies that promote coverage stability.

## Funding

Dr. Eliason was supported by the National Institute of Child Health and Human Development under grant award R00 HD111622. The funders had no role in the design and conduct of the study; collection, management, analysis, and interpretation of the data; preparation, review, or approval of the manuscript; and decision to submit the manuscript for publication. The content is solely the responsibility of the authors and does not necessarily represent the official views of the National Institutes of Health.

## Conflicts of Interest

The authors declare no conflicts of interest.

## Supporting information


**Appendix S1:** Supporting Appendix.
