# Supplementary material for: Children's Enrollment in Children's Health Insurance Program (CHIP) Coverage During the Medicaid Unwinding
Source: Health Serv Res. 2025 Dec 19;61(2):e70078. doi: 10.1111/1475-6773.70078 (PMC12932016; doi:10.1111/1475-6773.70078)
Supplement: Supplementary file 1 — Appendix S1: Supporting Appendix. [file HESR-61-1-s001.docx]

**Supplemental Appendix**

**Appendix A1.** Description of Data and Methods

**Appendix A2.** List of Study States and Comparison of State Eligibility and Structure Characteristics for Children

**Appendix A3.** Full Regression Results for Main Overall Adjusted Models

**Appendix A4.** Sensitivity Analyses Weighted by States’ Child Population Size

**Appendix A1. Description of Data and Methods**

*Data*

This study relied on state reports of monthly U.S. Centers for Medicare & Medicaid Services (CMS) enrollment data in Medicaid and CHIP. Separate CHIP enrollment by month and state was reported beginning April 1, 2023 as states were required to submit enrollment data to CMS to fulfill reporting requirements under section 1902(tt)(1) of the Social Security Act. Monthly state Medicaid and CHIP applications, eligibility determinations, and enrollment data are available on an ongoing basis, including reports of CHIP and Medicaid child enrollment. This CHIP and Medicaid child enrollment data reports all CHIP enrollment, including perinatal CHIP enrollment, and children enrolled in Medicaid. We subtracted CHIP enrollment from CHIP and Medicaid child enrollment data to identify children enrolled in Medicaid. CHIP enrollment numbers were derived from separate CHIP enrollment data or from CHIP and Medicaid child enrollment data. In states that offer perinatal CHIP coverage using the same CHIP program type as for children, we were unable to distinguish perinatal enrollees from child enrollees in the data. We therefore excluded the 16 states where children’s and perinatal CHIP enrollment could not be separated (AL, AR, CT, CA, CO, LA, MA, MO, NY, OR, SD, TN, TX, VA, WA, WI).

*Methods*

For each outcome, we estimated the following regression specification:

$$\Upsilon_{ST} = \beta_{1}{timefromApril2023}_{T} + \chi_{ST} +\in_{ST}$$

We estimated linear probability models with fixed effects for monthly indicators ranging from 0 to 17 for time since April 2023 ($\beta_{1}{timefromApril2023}_{T})$, reporting estimates for September 2024 (month 17) relative to the April 2023 baseline (month 0). $\Upsilon_{ST}$ represents our outcomes of interest. In adjusted models, $\chi_{ST}$ captures state fixed effects and covariates for CHIP structure type for children’s coverage, an indicator for which month the state began the Medicaid unwinding process, the unwinding strategy for prioritizing renewals (time-based approach, state-determined approach, or hybrid approach), estimated time to complete renewals (less than 9 months, 9 to less than 12 months, 12 to 14 months), policies for flagging potentially ineligible enrollees, whether states offered automated transfer to separate CHIP if Medicaid ex parte review confirmed CHIP eligibility, and the number of section 1902(e)(14) waivers used by states to address challenges related to Medicaid unwinding.

**Appendix A2. List of Study States and Comparison of State Eligibility and Structure Characteristics for Children**

In our data, we were unable to distinguish perinatal enrollees from child enrollees in states that offer perinatal CHIP using the same CHIP program type as children. As a result, we excluded all states where child and perinatal CHIP enrollment could not be separated. We additionally excluded Arizona, which did not report children’s enrollment, and Maryland, which changed their CHIP policies during the study. After these exclusions, our study included 32 states and the District of Columbia, listed below. Our study states captured the majority of states with M-CHIP structures and half of the states with combination CHIP. After our exclusions, our study states did not include either of the 2 states that offer only separate CHIP coverage (Connecticut and Washington). Our study states had comparable upper income eligibility limits for children’s Medicaid/CHIP (252% of the federal poverty level among study states vs 255% nationally).

| Study State | Medicaid/CHIP Upper Income Eligibility^a^ | State CHIP Structure Type^b^ |
| --- | --- | --- |
| Alaska | 208 | M-CHIP |
| Delaware | 217 | Combination |
| District of Columbia | 324 | M-CHIP |
| Florida | 215 | Combination |
| Georgia | 252 | Combination |
| Hawaii | 313 | M-CHIP |
| Idaho | 190 | Combination |
| Illinois | 318 | M-CHIP |
| Indiana | 255 | Combination |
| Iowa | 380 | Combination |
| Kansas | 2.55 | Combination |
| Kentucky | 218 | M-CHIP |
| Maine | 305 | M-CHIP |
| Michigan | 217 | M-CHIP |
| Minnesota | 288 | M-CHIP |
| Mississippi | 214 | Combination |
| Montana | 266 | Combination |
| Nebraska | 218 | M-CHIP |
| Nevada | 205 | Combination |
| New Hampshire | 323 | M-CHIP |
| New Jersey | 355 | Combination |
| New Mexico | 305 | M-CHIP |
| North Carolina | 216 | M-CHIP |
| North Dakota | 205 | M-CHIP |
| Ohio | 211 | M-CHIP |
| Oklahoma | 210 | M-CHIP |
| Pennsylvania | 319 | Combination |
| Rhode Island | 266 | M-CHIP |
| South Carolina | 213 | M-CHIP |
| Utah | 205 | Combination |
| Vermont | 317 | M-CHIP |
| West Virginia | 305 | Combination |
| Wyoming | 205 | M-CHIP |
| *Study States Overall:* | Median: 252 | Total: 19 M-CHIP; 14 Combination |
| *National Overall:* | Median: 255 | Total: 21 M-CHIP; 28 Combination; 2 Separate |

Notes: Eligibility is listed as a % of the federal poverty level.

^a^Source: Kaiser Family Foundation. CHIP Program Name and Type for Children-Only Coverage. State Health Facts. September 2024. https://www.kff.org/other/state-indicator/chip-program-name-and-type-for-children-only-coverage/

^b^Source: Kaiser Family Foundation. Medicaid/CHIP Upper Income Eligibility Limits for Children, 2024. Trends in Income Eligibility Limits for Children. May 2024. https://www.kff.org/medicaid/state-indicator/medicaidchip-upper-income-eligibility-limits-for-children/

**Appendix A3. Full Regression Results for Main Overall Adjusted Models**

This table presents the full regression results for the main overall adjusted models for the change in children’s enrollment in Medicaid and CHIP from April 2023 to September 2024, 17 months into the Medicaid unwinding, with coefficients presented for each month relative to April 2023 and covariates for state CHIP and Medicaid characteristics, unwinding policies, and state fixed effects.

| Outcome: | Average Number of Children Enrolled in CHIP | Average Number of Children Enrolled in Medicaid | Average Proportion of Children Enrolled in CHIP | Average Proportion of Children Enrolled in Medicaid |
| --- | --- | --- | --- | --- |
|  |  |  |  |  |
| Time since April 2023 | | | | |
| 1 month (May 2023) | -161 | 12,458 | -0.1 | 0.2 |
| (95% CI) | (-3,083 to 2,761) | (-3,529 to 28,445) | (-0.4 to 0.2) | (-0.3 to 0.8) |
| *P*-value | 0.911 | 0.122 | 0.543 | 0.389 |
| 2 months (June 2023) | -401 | 6,267 | -0.4 | -0.7 |
|  | (-3,911 to 3,108) | (-6,685 to 19,218) | (-0.9 to 0.2) | (-1.5 to 0.2) |
|  | 0.817 | 0.332 | 0.165 | 0.107 |
| 3 months (July 2023) | -256 | 1,961 | -0.5 | -1.4 |
|  | (-4,766 to 4,254) | (-15,047 to 18,970) | (-1.1 to 0.2) | (-2.5 to -0.3) |
|  | 0.909 | 0.816 | 0.133 | 0.016 |
| 4 months (Aug 2023) | 20 | -9,490 | -0.6 | -2.3 |
|  | (-4,915 to 4,955) | (-29,678 to 10,697) | (-1.2 to 0.1) | (-3.5 to -1.1) |
|  | 0.993 | 0.345 | 0.107 | <0.001 |
| 5 months (Sep 2023) | 549 | -18,460 | -0.5 | -3.0 |
|  | (-4,958 to 6,056) | (-43,408 to 6,487) | (-1.2 to 0.2) | (-4.4 to -1.6) |
|  | 0.840 | 0.142 | 0.169 | <0.001 |
| 6 months (Oct 2023) | 1,207 | -24,354 | -0.5 | -3.3 |
|  | (-4,636 to 7,050) | (-51,340 to 2,633) | (-1.2 to 0.3) | (-4.7 to -1.9) |
|  | 0.677 | 0.075 | 0.213 | <0.001 |
| 7 months (Nov 2023) | 1,291 | -35,058 | -0.5 | -3.9 |
|  | (-5,076 to 7,657) | (-69,256 to -861) | (-1.2 to 0.3) | (-5.4 to -2.5) |
|  | 0.682 | 0.045 | 0.208 | <0.001 |
| 8 months (Dec 2023) | 1,919 | -42,676 | -0.4 | -4.4 |
|  | (-4,827 to 8,666) | (-82,396 to -2,956) | (-1.2 to 0.4) | (-6.0 to -2.8) |
|  | 0.566 | 0.036 | 0.275 | <0.001 |
| 9 months (Jan 2024) | 1,774 | -46,640 | -0.4 | -4.7 |
|  | (-6,020 to 9,568) | (-89,775 to -3,506) | (-1.2 to 0.5) | (-6.3 to -3.1) |
|  | 0.646 | 0.035 | 0.361 | <0.001 |
| 10 months (Feb 2024) | 1,483 | -49,282 | -0.4 | -5.1 |
|  | (-7,124 to 10,089) | (-92,219 to -6,345) | (-1.3 to 0.5) | (-6.7 to -3.5) |
|  | 0.728 | 0.026 | 0.357 | <0.001 |
| 11 months (Mar 2024) | 1,593 | -52,030 | -0.4 | -5.4 |
|  | (-7,386 to 10,573) | (-95,732 to -8,328) | (-1.3 to 0.5) | (-7.0 to -3.8) |
|  | 0.720 | 0.021 | 0.401 | <0.001 |
| 12 months (Apr 2024) | 392 | -54,796 | -0.5 | -5.7 |
|  | (-8,971 to 9,754) | (-99,467 to -10,125) | (-1.4 to 0.4) | (-7.2 to -4.1) |
|  | 0.933 | 0.018 | 0.282 | <0.001 |
| 13 months (May 2024) | -497 | -57,708 | -0.6 | -6.0 |
|  | (-10,068 to 9,075) | (-103,153 to -12,263) | (-1.5 to 0.4) | (-7.6 to -4.4) |
|  | 0.916 | 0.014 | 0.238 | <0.001 |
| 14 months (June 2024) | -968 | -60,494 | -0.6 | -6.4 |
|  | (-10,722 to 8,786) | (-106,568 to -14,419) | (-1.6 to 0.4) | (-8.0 to -4.7) |
|  | 0.841 | 0.012 | 0.222 | <0.001 |
| 15 months (July 2024) | -1,321 | -61,921 | -0.6 | -6.5 |
|  | (-11,220 to 8,578) | (-107,763 to -16,080) | (-1.5 to 0.4) | (-8.1 to -4.9) |
|  | 0.787 | 0.010 | 0.257 | <0.001 |
| 16 months (Aug 2024) | -1,123 | -61,225 | -0.5 | -6.4 |
|  | (-11,245 to 8,999) | (-106,759 to -15,690) | (-1.5 to 0.5) | (-8.0 to -4.9) |
|  | 0.823 | 0.010 | 0.299 | <0.001 |
| 17 months (Sep 2024) | -740 | -62,032 | -0.5 | -6.5 |
|  | (-10,815 to 9,336) | (-108,018 to -16,045) | (-1.4 to 0.5) | (-8.1 to -5.0) |
|  | 0.882 | 0.010 | 0.327 | <0.001 |
| CHIP Structure Type |  |  |  |  |
| Combination CHIP | 21,715 | 173,562 | 2.9 | 5.6 |
|  | (3,311 to 40,118) | (93,186 to 253,939) | (1.1 to 4.7) | (0.3 to 10.9) |
|  | 0.022 | <0.001 | 0.003 | 0.040 |
| First Month of Medicaid Unwinding | | | | |
| May 2023 | -58,015 | 67,271 | -4.3 | 16.5 |
|  | (-75,927 to -40,102) | (-52,182 to 186,725) | (-6.1 to -2.5) | (10.9 to 22.0) |
|  | <0.001 | 0.260 | <0.001 | <0.001 |
| June 2023 | -67,452 | 74,814 | -6.8 | 15.5 |
|  | (-87,318 to -47,586) | (-54,410 to 204,038) | (-8.6 to -4.9) | (9.3 to 21.7) |
|  | <0.001 | 0.247 | <0.001 | <0.001 |
| July 2023 | 170,080 | 533,828 | -1.1 | -5.4 |
|  | (144,434 to 195,725) | (412,822 to 654,835) | (-4.0 to 1.9) | (-13.9 to 3.1) |
|  | <0.001 | <0.001 | 0.467 | 0.207 |
| Aug 2023 | -59,856 | 141,452 | -4.1 | 14.6 |
|  | (-78,999 to -40,712) | (9,853 to 273,050) | (-6.4 to -1.8) | (8.6 to 20.7) |
|  | <0.001 | 0.036 | 0.001 | <0.001 |
| Unwinding Strategy for Prioritizing Renewals | | | | |
| Time-based approach | 6,872 | -506,219 | -0.9 | -10.7 |
|  | (-9,860 to 23,605) | (-613,185 to -399,253) | (-2.2 to 0.4) | (-15.3 to -6.2) |
|  | 0.409 | <0.001 | 0.167 | <0.001 |
| Population-based approach | 28,718 | -485,861 | -0.9 | -11.7 |
|  | (12,759 to 44,677) | (-584,861 to -386,860) | (-2.0 to 0.2) | (-15.4 to -8.0) |
|  | 0.001 | <0.001 | 0.112 | <0.001 |
| State-determined approach | 25,388 | -613,364 | -0.6 | -9.5 |
|  | (1,227 to 49,548) | (-778,750 to -447,979) | (-2.3 to 1.1) | (-15.1 to -4.0) |
|  | 0.040 | <0.001 | 0.461 | 0.001 |
| Hybrid approach | 16,185 | -542,758 | -0.6 | -9.3 |
|  | (-7,418 to 39,788) | (-691,990 to -393,527) | (-2.3 to 1.1) | (-14.6 to -4.0) |
|  | 0.172 | <0.001 | 0.461 | 0.001 |
| Not recorded | 103,156 | -576,552 | 4.6 | -1.8 |
|  | (75,103 to 131,210) | (-799,439 to -353,666) | (2.5 to 6.7) | (-9.1 to 5.5) |
|  | <0.001 | <0.001 | <0.001 | 0.627 |
| Estimated Time to Complete Renewals | | | | |
| 12-14 Months | 40,630 | 9,312 | 3.2 | 3.0 |
|  | (27,817 to 53,443) | (-74,775 to 93,400) | (2.8 to 3.7) | (0.7 to 5.3) |
|  | <0.001 | 0.823 | <0.001 | 0.013 |
| Flagging Potentially Ineligible Enrollees | | | | |
| Yes | -22,261 | 79,659 | -1.2 | -2.7 |
|  | (-36,786 to -7,737) | (-39,306 to 198,624) | (-2.5 to 0.0) | (-6.9 to 1.4) |
|  | 0.004 | 0.182 | 0.058 | 0.187 |
| Automated Transfer to Separate CHIP from Medicaid Ex Parte Review | | | | |
| Yes | 4,641 | 125,141 | 0.2 | 1.5 |
|  | (-12,128 to 21,411) | (5,055 to 245,228) | (-1.5 to 2.0) | (-4.4 to 7.5) |
|  | 0.577 | 0.042 | 0.778 | 0.603 |
| N/A | -22,961 | 207,687 | -1.3 | 1.1 |
|  | (-45,757 to -165) | (20,640 to 394,733) | (-3.5 to 0.8) | (-6.4 to 8.7) |
|  | 0.048 | 0.031 | 0.221 | 0.760 |
| Number of Section 1902(e)(14) Waivers | | | | |
| 2 | -27,823 | 315,439 | 0.4 | 4.9 |
|  | (-49,238 to -6,407) | (120,063 to 510,814) | (-1.5 to 2.2) | (-2.7 to 12.6) |
|  | 0.013 | 0.002 | 0.674 | 0.200 |
| 3 | -17,468 | 296,550 | -0.2 | 8.6 |
|  | (-41,192 to 6,257) | (98,599 to 494,502) | (-2.2 to 1.8) | (0.9 to 16.3) |
|  | 0.143 | 0.005 | 0.826 | 0.030 |
| 4 | -38,684 | 447,065 | -0.5 | 7.9 |
|  | (-55,110 to -22,259) | (363,330 to 530,801) | (-2.4 to 1.5) | (2.1 to 13.7) |
|  | <0.001 | <0.001 | 0.634 | 0.009 |
| 5 | -58,313 | 393,938 | -1.6 | 8.1 |
|  | (-73,116 to -43,509) | (310,442 to 477,434) | (-3.3 to 0.2) | (2.8 to 13.4) |
|  | <0.001 | <0.001 | 0.077 | 0.004 |
| 6 | -35,437 | 331,156 | -0.7 | 4.5 |
|  | (-56,183 to -14,690) | (142,336 to 519,975) | (-2.6 to 1.1) | (-3.0 to 12.1) |
|  | 0.001 | 0.001 | 0.411 | 0.231 |
| 7 | -96,934 | 310,732 | -3.5 | 1.2 |
|  | (-115,594 to -78,274) | (215,075 to 406,388) | (-5.3 to -1.7) | (-4.1 to 6.5) |
|  | <0.001 | <0.001 | <0.001 | 0.648 |
| 8 | -49,428 | 399,413 | -0.7 | 5.7 |
|  | (-67,024 to -31,832) | (272,232 to 526,595) | (-2.7 to 1.2) | (-0.5 to 11.9) |
|  | <0.001 | <0.001 | 0.463 | 0.072 |
| 9 | -48,807 | 431,589 | -1.6 | 8.3 |
|  | (-59,663 to -37,950) | (348,990 to 514,188) | (-2.7 to -0.5) | (4.4 to 12.3) |
|  | <0.001 | <0.001 | 0.006 | <0.001 |
| 10 | -29,945 | 361,219 | 0.3 | 7.4 |
|  | (-49,170 to -10,720) | (195,899 to 526,540) | (-2.2 to 2.8) | (0.0 to 14.7) |
|  | 0.003 | <0.001 | 0.797 | 0.049 |
| 11 | 23,568 | 204,186 | 1.7 | 11.6 |
|  | (-12,142 to 59,277) | (-100,594 to 508,966) | (-1.4 to 4.8) | (0.8 to 22.3) |
|  | 0.188 | 0.182 | 0.278 | 0.036 |
| 12 | -30,482 | 329,724 | -0.3 | 4.2 |
|  | (-46,902 to -14,063) | (210,076 to 449,373) | (-2.0 to 1.4) | (-1.7 to 10.1) |
|  | 0.001 | <0.001 | 0.726 | 0.159 |
| 13 | -49,758 | 358,111 | -2.1 | 6.4 |
|  | (-68,449 to -31,068) | (204,761 to 511,462) | (-3.9 to -0.3) | (-0.4 to 13.1) |
|  | <0.001 | <0.001 | 0.026 | 0.064 |
| 14 | -23,632 | 313,206 | -0.4 | 8.2 |
|  | (-47,137 to -127) | (122,084 to 504,328) | (-2.7 to 1.9) | (0.2 to 16.3) |
|  | 0.049 | 0.002 | 0.729 | 0.045 |
| 15 | -28,055 | 276,223 | -0.8 | 9.7 |
|  | (-54,090 to -2,020) | (51,774 to 500,673) | (-3.2 to 1.6) | (1.2 to 18.3) |
|  | 0.036 | 0.017 | 0.496 | 0.027 |
| State Fixed Effects | | | | |
| Delaware | -33,419 | -107,559 | -6.7 | -2.5 |
|  | (-47,578 to -19,260) | (-129,672 to -85,446) | (-8.6 to -4.8) | (-7.5 to 2.6) |
|  | <0.001 | <0.001 | <0.001 | 0.327 |
| District of Columbia | -5,310 | 60,244 | 5.5 | 16.3 |
|  | (-17,839 to 7,218) | (-36,691 to 157,179) | (5.0 to 5.9) | (13.5 to 19.1) |
|  | 0.394 | 0.215 | <0.001 | <0.001 |
| Florida | 47,657 | 2,797,725 | -8.0 | 11.0 |
|  | (33,611 to 61,704) | (2,781,495 to 2,813,956) | (-9.8 to -6.3) | (6.2 to 15.9) |
|  | <0.001 | <0.001 | <0.001 | <0.001 |
| Georgia | 274,280 | 1,214,215 | 2.4 | -1.1 |
|  | (260,245 to 288,315) | (1,197,911 to 1,230,518) | (0.6 to 4.2) | (-6.0 to 3.7) |
|  | <0.001 | <0.001 | 0.011 | 0.638 |
| Hawaii | 19,994 | 86,695 | 2.0 | -2.9 |
|  | (6,299 to 33,690) | (-24,725 to 198,116) | (1.5 to 2.4) | (-6.2 to 0.4) |
|  | 0.006 | 0.123 | <0.001 | 0.085 |
| Idaho | -65,537 | 261,213 | -6.9 | -24.9 |
|  | (-98,469 to -32,605) | (-39,945 to 562,371) | (-9.5 to -4.3) | (-35.5 to -14.4) |
|  | <0.001 | 0.087 | <0.001 | <0.001 |
| Illinois | 290,555 | 1,180,890 | 4.5 | -6.2 |
|  | (278,551 to 302,560) | (1,101,781 to 1,259,999) | (4.1 to 4.9) | (-8.4 to -4.0) |
|  | <0.001 | <0.001 | <0.001 | <0.001 |
| Indiana | 96,203 | 525,103 | -1.4 | -10.4 |
|  | (82,168 to 110,238) | (508,800 to 541,407) | (-3.2 to 0.4) | (-15.3 to -5.5) |
|  | <0.001 | <0.001 | 0.127 | <0.001 |
| Iowa | 29,012 | 95,158 | -0.5 | -14.0 |
|  | (14,966 to 43,059) | (78,928 to 111,389) | (-2.3 to 1.3) | (-18.9 to -9.2) |
|  | <0.001 | <0.001 | 0.552 | <0.001 |
| Kansas | 48,192 | -67,491 | -0.3 | -17.4 |
|  | (27,805 to 68,580) | (-189,261 to 54,280) | (-2.6 to 2.1) | (-23.8 to -11.0) |
|  | <0.001 | 0.267 | 0.818 | <0.001 |
| Kentucky | 112,620 | 500,213 | 6.2 | 1.9 |
|  | (99,320 to 125,920) | (412,803 to 587,623) | (5.2 to 7.2) | (-2.6 to 6.4) |
|  | <0.001 | <0.001 | <0.001 | 0.386 |
| Maine | -1,841 | 74,180 | 0.2 | -1.8 |
|  | (-13,847 to 10,165) | (-5,070 to 153,431) | (-0.6 to 1.0) | (-4.2 to 0.5) |
|  | 0.757 | 0.066 | 0.613 | 0.126 |
| Michigan | 127,610 | 915,450 | -0.1 | -5.8 |
|  | (114,931 to 140,289) | (822,490 to 1,008,409) | (-0.5 to 0.3) | (-8.5 to -3.1) |
|  | <0.001 | <0.001 | 0.643 | <0.001 |
| Minnesota | -17,264 | 575,768 | -7.0 | -2.6 |
|  | (-29,233 to -5,296) | (495,644 to 655,892) | (-7.6 to -6.4) | (-5.0 to -0.1) |
|  | 0.006 | <0.001 | <0.001 | 0.043 |
| Mississippi | 37,249 | 163,816 | 1.6 | 1.3 |
|  | (23,172 to 51,326) | (137,816 to 189,817) | (-0.2 to 3.4) | (-3.7 to 6.2) |
|  | <0.001 | <0.001 | 0.077 | 0.608 |
| Montana | -11,701 | -121,358 | 2.2 | -13.6 |
|  | (-25,747 to 2,346) | (-137,588 to -105,127) | (0.4 to 4.0) | (-18.5 to -8.8) |
|  | 0.099 | <0.001 | 0.018 | <0.001 |
| Nebraska | 16,540 | 152,526 | 1.4 | -17.4 |
|  | (3,026 to 30,053) | (60,756 to 244,296) | (0.7 to 2.2) | (-20.1 to -14.7) |
|  | 0.018 | 0.002 | <0.001 | <0.001 |
| Nevada | 11,021 | 112,505 | -2.2 | -11.6 |
|  | (-3,964 to 26,005) | (45,641 to 179,369) | (-4.0 to -0.4) | (-16.9 to -6.3) |
|  | 0.144 | 0.002 | 0.019 | <0.001 |
| New Hampshire | 58,015 | -67,271 | 4.3 | -16.5 |
|  | (40,102 to 75,927) | (-186,725 to 52,182) | (2.5 to 6.1) | (-22.0 to -10.9) |
|  | <0.001 | 0.260 | <0.001 | <0.001 |
| New Jersey | 228,094 | 466,557 | 3.2 | -21.8 |
|  | (215,303 to 240,885) | (452,559 to 480,555) | (1.6 to 4.9) | (-26.3 to -17.4) |
|  | <0.001 | <0.001 | <0.001 | <0.001 |
| New Mexico | 27,967 | 286,305 | 3.4 | 18.5 |
|  | (14,667 to 41,267) | (198,896 to 373,715) | (2.4 to 4.5) | (14.0 to 23.0) |
|  | <0.001 | <0.001 | <0.001 | <0.001 |
| North Carolina | 287,231 | 1,047,101 | 6.0 | -5.2 |
|  | (275,390 to 299,071) | (968,775 to 1,125,428) | (5.6 to 6.4) | (-7.4 to -3.1) |
|  | <0.001 | <0.001 | <0.001 | <0.001 |
| North Dakota | -18,724 | 1,162 | -6.1 | -22.9 |
|  | (-30,683 to -6,765) | (-77,810 to 80,133) | (-7.0 to -5.2) | (-25.4 to -20.5) |
|  | 0.003 | 0.976 | <0.001 | <0.001 |
| Ohio | 235,202 | 1,059,462 | 2.7 | -9.3 |
|  | (223,161 to 247,243) | (979,972 to 1,138,953) | (2.3 to 3.1) | (-11.4 to -7.1) |
|  | <0.001 | <0.001 | <0.001 | <0.001 |
| Oklahoma | 121,900 | 458,968 | 7.4 | 0.5 |
|  | (109,859 to 133,941) | (379,478 to 538,459) | (7.0 to 7.8) | (-1.7 to 2.6) |
|  | <0.001 | <0.001 | <0.001 | 0.647 |
| Pennsylvania | 216,681 | 1,072,276 | -0.4 | -8.1 |
|  | (198,319 to 235,044) | (992,077 to 1,152,475) | (-2.2 to 1.4) | (-13.4 to -2.8) |
|  | <0.001 | <0.001 | 0.648 | 0.004 |
| Rhode Island | 10,102 | 30,680 | 8.7 | -4.6 |
|  | (-5,080 to 25,284) | (-48,505 to 109,864) | (7.5 to 10.0) | (-8.6 to -0.7) |
|  | 0.185 | 0.436 | <0.001 | 0.023 |
| South Carolina | 95,244 | 563,040 | 2.7 | 3.6 |
|  | (81,871 to 108,618) | (457,035 to 669,044) | (2.3 to 3.1) | (0.5 to 6.8) |
|  | <0.001 | <0.001 | <0.001 | 0.024 |
| Utah | -3,970 | -33,676 | -6.7 | -35.1 |
|  | (-18,405 to 10,465) | (-63,656 to -3,697) | (-8.7 to -4.6) | (-40.5 to -29.8) |
|  | 0.579 | 0.029 | <0.001 | <0.001 |
| Vermont | -9,437 | 7,543 | -2.5 | -1.0 |
|  | (-24,580 to 5,705) | (-71,328 to 86,413) | (-3.7 to -1.2) | (-4.9 to 3.0) |
|  | 0.213 | 0.847 | <0.001 | 0.616 |
| Wyoming | -12,289 | 20,352 | -3.1 | -14.0 |
|  | (-25,641 to 1,063) | (-83,045 to 123,749) | (-3.5 to -2.8) | (-17.2 to -10.8) |
|  | 0.070 | 0.691 | <0.001 | <0.001 |

Notes: Authors’ analysis of U.S. Centers for Medicare & Medicaid Services enrollment data in Medicaid and CHIP, April 2023 to September 2024. CHIP is the Children’s Health Insurance Program.

**Appendix A4. Sensitivity Analyses Weighted by States’ Child Population Size**

As a sensitivity analysis, we estimated models weighted by states’ child population size, using population data from the U.S. Census Bureau. In these models, some effect sizes for the overall declines in children’s Medicaid enrollment were larger, but all estimates had confidence intervals that overlapped with those in the main models. The only change to statistical significance was for the average number of children enrolled in Medicaid among states with combination CHIP structures in adjusted models. In analyses weighted by population, the declines in the number of children enrolled in Medicaid were not statistically significant (242,874 fewer children, 95% CI: -493,986 to 8,237; compared to 102,518 fewer children, 95% CI: −200,830 to −4,206 in main models) among states with combination CHIP structures.

| Outcome | Start of FFCRA Medicaid Unwinding, April 2023 | 17 Months into Medicaid Unwinding, September 2024 | Unadjusted Change  (95% CI) | *P-*Value | Adjusted Change  (95% CI) | *P-*Value |
| --- | --- | --- | --- | --- | --- | --- |
| *Overall* | | | | | | |
| Average Number of Children Enrolled in CHIP | 166,909 | 170,509 | 3,599  (-21,660 to 28,858) | 0.773 | 8,511  (-20,301 to 37,323) | 0.552 |
| Average Number of Children Enrolled in Medicaid | 1,093,786 | 911,717 | -182,069  (-328,630 to -35,508) | 0.017 | -171,191  (-330,828 to -11,553) | 0.036 |
| Average Proportion of Children Enrolled in CHIP | 8.8 | 8.7 | -0.1  (-1.0 to 0.7) | 0.744 | -0.1  (-1.2 to 0.9) | 0.790 |
| Average Proportion of Children Enrolled in Medicaid | 49.9 | 42.0 | -7.9  (-11.0 to -4.8) | <0.001 | -7.5  (-10.6 to -4.5) | <0.001 |
| *States with M-CHIP Structures* | | | | | | |
| Average Number of Children Enrolled in CHIP | 176,710 | 170,098 | -6,612  (-29,918 to 16,695) | 0.559 | -8,767  (-27,532 to 9,997) | 0.339 |
| Average Number of Children Enrolled in Medicaid | 825,527 | 740,143 | -85,383  (-133,050 to -37,717) | 0.001 | -64,652  (-108,965 to -20,338) | 0.007 |
| Average Proportion of Children Enrolled in CHIP | 9.6 | 9.2 | -0.5  (-1.7 to 0.7) | 0.405 | -0.8  (-2.2 to 0.6) | 0.245 |
| Average Proportion of Children Enrolled in Medicaid | 47.9 | 42.8 | -5.1  (-7.3 to -2.9) | <0.001 | -4.5  (-6.2 to -2.8) | <0.001 |
| *States with Combination CHIP Structures* | | | | | | |
| Average Number of Children Enrolled in CHIP | 157,819 | 170,887 | 13,068  (-32,366 to 58,503) | 0.545 | 21,005  (-18,926 to 60,936) | 0.276 |
| Average Number of Children Enrolled in Medicaid | 1,342,614 | 1,069,758 | -272,856  (-539,256 to -6,457) | 0.045 | -242,874  (-493,986 to 8,237) | 0.057 |
| Average Proportion of Children Enrolled in CHIP | 8.1 | 8.3 | 0.2  (-1.2 to 1.5) | 0.785 | 0.3  (-1.1 to 1.7) | 0.673 |
| Average Proportion of Children Enrolled in Medicaid | 51.7 | 41.3 | -10.5  (-15.3 to -5.6) | <0.001 | -9.7  (-13.8 to -5.7) | <0.001 |

Notes: Authors’ analysis of U.S. Centers for Medicare & Medicaid Services enrollment data in Medicaid and CHIP, April 2023 to September 2024. CHIP is the Children’s Health Insurance Program.
